# Sustained improvements in health status and work productivity with bimekizumab in psoriatic arthritis: 2-year results from two phase 3 studies

**DOI:** 10.1093/rap/rkag009

**Published:** 2026-01-25

**Authors:** William Tillett, Laure Gossec, M Elaine Husni, Akimichi Morita, Barbara Ink, Rajan Bajracharya, Patrick Healy, Michael F Mørup, Jessica A Walsh

**Affiliations:** Royal National Hospital of Rheumatic Diseases, Bath, UK; Department of Life Sciences, Centre for Therapeutic Innovation, University of Bath, Bath, UK; Sorbonne Université, INSERM, Institut Pierre Louis d‘Epidémiologie et de Santé Publique, Paris, France; Rheumatology Department, AP-HP, Pitié-Salpêtrière Hospital, Paris, France; Department of Rheumatic and Immunologic Diseases, Cleveland Clinic, Cleveland, OH, USA; Department of Geriatric and Environmental Dermatology, Nagoya City University Graduate School of Medical Sciences, Nagoya, Japan; UCB, Slough, UK; UCB, Slough, UK; UCB, Morrisville, NC, USA; UCB, Copenhagen, Denmark; Division of Rheumatology, Salt Lake City Veterans Affairs Health and University of Utah Health, Salt Lake City, UT, USA

**Keywords:** bimekizumab, psoriatic arthritis, health status, work productivity, cost savings, IL-17

## Abstract

**Objectives:**

To assess the 2-year impact of bimekizumab on health status, work productivity and indirect cost savings in patients with active PsA.

**Methods:**

BE OPTIMAL [NCT03895203; biologic disease-modifying anti-rheumatic drug (bDMARD)-naïve] and BE COMPLETE [NCT03896581; tumor necrosis factor inhibitor inadequate response/intolerance (TNFi-IR)] assessed subcutaneous bimekizumab 160 mg every 4 weeks. BE OPTIMAL week 52/BE COMPLETE week 16 completers could enter BE VITAL (NCT04009499; open-label extension), where all patients received bimekizumab. Outcomes reported for placebo- and bimekizumab-randomized patients to year 2 were changes in health status (EQ-5D-3L) and work productivity [Work Productivity and Activity Impairment Questionnaire—Specific Health Problem v2.0 (WPAI-SHP)], associations between disease control criteria and WPAI-SHP, and annualized indirect cost savings (estimated by multiplying 2022 average wages by improvements in overall work impairment). Missing data were imputed using multiple (continuous) or non-responder (binary) imputation.

**Results:**

Among 712 bDMARD-naïve and 400 TNFi-IR patients, improvements from baseline in mean EQ-5D-3L visual analog scale scores were sustained to year 2, with scores improving by 24.6–38.9%. A minimal clinically important difference in overall work impairment was achieved by 51.1–64.8% of patients at year 1, and this was largely sustained to year 2 (44.3–51.6%). The greatest work productivity improvements were observed in patients who achieved stringent disease control across both musculoskeletal and skin domains. In Europe, indirect cost savings at year 1 (US$7965–11 517) were sustained to year 2 (US$7789–12 018); trends were similar in the USA and Japan.

**Conclusion:**

Bimekizumab treatment resulted in sustained improvements in health status and work productivity to year 2, with substantial potential for long-term indirect cost savings, regardless of prior TNFi experience.

## Graphical Abstract

**Figure:**
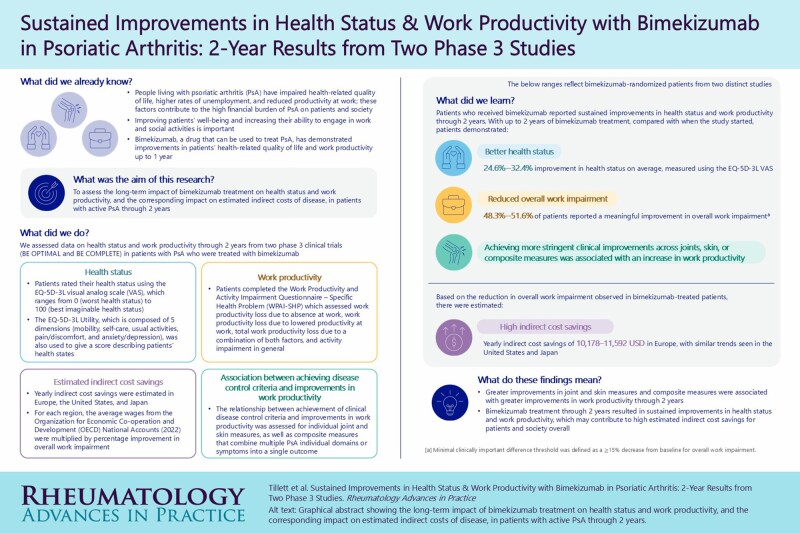

Key messages

- Bimekizumab demonstrated sustained improvements in health status and work productivity to 2 years, regardless of prior TNFi experience.
- Patients with PsA who achieved more stringent disease control had greater improvements in work productivity.
- Work productivity improvements with bimekizumab contributed to high estimated indirect cost savings, which could benefit patients and society overall.

## Introduction

PsA is a chronic, progressive inflammatory condition occurring in up to 30% of patients with psoriasis that affects multiple tissues and clinical domains, including peripheral and axial joints, skin, nails and entheses [1–3]. Symptoms include joint swelling, skin disease, pain and fatigue, which can endure despite treatment [4]. Persistent active disease can lead to progressive structural joint damage and disability [4, 5]. Moreover, unresolved inflammation is associated with comorbidities, including uveitis, cardiovascular disease and subclinical bowel inflammation, contributing to overall disease burden [4].

These symptoms have detrimental effects on patients’ health-related quality of life (HRQoL) [2, 3, 6], including workplace and household productivity [6, 7]. For instance, patients with PsA experience work absences, reduced work capacity, productivity loss and, particularly among those with worse physical function, higher levels of unemployment [2, 7]. Nevertheless, patients with chronic diseases financially benefit from working while also gaining a more positive self-identity, sense of normalcy and the feeling of being a productive member of society [8, 9].

PsA exerts a high economic burden upon patients and society [10, 11]. Indirect costs related to productivity loss, paid work absences, occupational disability, early retirement and unemployment are higher when considering patients with PsA as compared with the general population [12–15]. A systematic literature review estimated that the average annual indirect costs of PsA, in US dollars (US$) and 2013 prices, range from US$1694 to US$12 318 (friction cost approach) or US$1751 to US$50 271 (human capital approach) [16]. While treatments that improve disease control are associated with improvements in patients’ HRQoL, there can be a gap between symptom relief and improvements in functional status; the latter must occur before there can be an improvement in work productivity [17].

Bimekizumab is a monoclonal IgG1 antibody that selectively inhibits IL-17F in addition to IL-17A [18, 19]. Bimekizumab has demonstrated high levels of response in threshold-based clinical outcomes, including minimal disease activity (MDA), and sustained improvements in patient-reported symptoms, including pain and fatigue, up to 2 years among patients with active PsA who were biologic DMARD (bDMARD)-naïve or had inadequate response or intolerance to TNF inhibitors (TNFi-IR) [20]. Bimekizumab also demonstrated sustained improvements in HRQoL and work productivity up to 1 year in patients with active PsA who were bDMARD-naïve or TNFi-IR [21].

Here, we report the long-term impact of bimekizumab on health status and work productivity for bDMARD-naïve and TNFi-IR patients with PsA receiving an additional year of treatment, up to 2 years. For the first time, an indirect cost analysis based on improved overall work impairment up to 2 years has been conducted.

## Methods

### Study designs and patients

Full methodological details of the phase 3, multicenter BE OPTIMAL (bDMARD-naïve patients; NCT03895203) and BE COMPLETE (TNFi-IR patients; NCT03896581) studies have been reported previously [22, 23]. In brief, both were double-blind and placebo-controlled to 16 weeks, assessing the efficacy and safety of bimekizumab in patients with active PsA who were bDMARD-naïve or TNFi-IR; study designs are shown in Supplementary Fig. S1.

BE OPTIMAL patients were randomized 3:2:1 to receive s.c. bimekizumab 160 mg every 4 weeks (Q4W), placebo or reference (s.c. adalimumab 40 mg Q2W; data not reported here). At week 16, patients receiving placebo switched to bimekizumab (placebo/bimekizumab). Those randomized to bimekizumab or adalimumab continued their dosing until week 52. BE COMPLETE patients were randomized 2:1 to s.c. bimekizumab 160 mg Q4W or placebo. Patients completing week 52 of BE OPTIMAL or week 16 of BE COMPLETE could enter BE VITAL [NCT04009499; open-label extension (OLE)], in which all patients received bimekizumab 160 mg Q4W.

Change from baseline (CfB), associations and responder rate data up to 2 years are reported for placebo/bimekizumab and bimekizumab-randomized patients. In BE VITAL, the interval between assessment timepoints was kept consistent; however, due to differences in entry weeks among patients in BE OPTIMAL *vs* BE COMPLETE, this approach resulted in variations in the assessment timepoints between the two studies. Consequently, the 2-year time point corresponds to week 104 in BE OPTIMAL and week 88 in BE COMPLETE.

The full schedule of assessments is presented in Supplementary Table S1 and summarized below.

### Health status

Health status was assessed using the European Quality of Life 5-Dimensions 3-Level (EQ-5D-3L) questionnaire visual analog scale (VAS) and EQ-5D-3L utility (UK tariff; commonly used in similar studies) [7], for all patients. For the EQ-5D-3L VAS, patients rate their health status on a scale of 0–100 (0 and 100 representing the worst and best imaginable health status, respectively). For the EQ-5D-3L utility (UK tariff), patients assess their health across five dimensions (mobility, self-care, usual activities, pain/discomfort, anxiety/depression) and three levels of severity (no problems, some problems, extreme problems); these are used together to generate a health state profile ranging from <0 (0 is a health state equivalent to death; negative values are valued as worse than death) to 1 (perfect health) [24, 25].

The EQ-5D-3L VAS was assessed at baseline in both studies and subsequently at weeks 4, 16, 24, 36, 52, 76 and 104 (BE OPTIMAL) and weeks 4, 12, 16, 40, 68 and 88 (BE COMPLETE).

### Work productivity

Work productivity was assessed using the Work Productivity and Activity Impairment Questionnaire—Specific Health Problem v2.0 (WPAI-SHP), adapted for PsA [26]. The WPAI-SHP consists of six questions, of which five are regrouped into four domains. Work time missed (absenteeism), impairment at work (presenteeism) and overall work impairment (sum of absenteeism and presenteeism) were assessed in employed patients. Activity impairment outside work attributable to PsA was assessed in all patients, regardless of employment status.

The WPAI-SHP minimal clinically important difference (MCID) threshold was defined as a ≥15% decrease from baseline for overall work impairment, ≥20% decrease from baseline for presenteeism and ≥20% decrease from baseline for activity impairment [26].

The association between clinical disease control measures and improvements in WPAI-SHP domains were examined, including a <20% to 70% improvement from baseline in ACR (<ACR20–ACR70), Disease Activity Index for Psoriatic Arthritis [DAPSA; high disease activity (HDA), moderate disease activity (MoDA), low disease activity/remission (LDA/REM)] and minimal disease activity (MDA; non-responder and responder) in all patients.

In patients with psoriasis affecting ≥3% of the body surface area (BSA) at baseline, the association between improvements in WPAI-SHP domains and simultaneous achievement of ACR50 and 100% improvement in the Psoriasis Area Severity Index (ACR50 + PASI100) were examined.

WPAI-SHP domains were assessed at baseline in both studies and subsequently at weeks 16, 24, 52, 76 and 104 (BE OPTIMAL) and weeks 12, 16, 40, 68 and 88 (BE COMPLETE).

### Cost analysis

Annualized indirect cost savings from improved overall work impairment in employed patients are reported. Indirect cost savings were estimated by multiplying the Organization for Economic Co-operation and Development (OECD)-reported national accounts average wages for 2022 for Europe (including France, Germany, Italy, Spain and the UK), the USA and Japan [27] by the percentage CfB in WPAI-SHP overall work impairment at week 52/40 and week 104/88. This method has been applied in other, similar analyses [28]. OECD national accounts wages include both full-time and part-time workers at a full-time equivalent rate and include average earnings from secondary jobs for workers with two or more jobs [29]. All values are presented in US dollars.

### Statistical analysis

Statistical powering and sample size determination for BE OPTIMAL and BE COMPLETE have been reported elsewhere [22, 23]. Non-responder imputation (NRI) was used to impute missing data for binary endpoints and multiple imputation (MI) was used to impute missing data for continuous outcomes for all patients to 2 years. Data were imputed using baseline patient numbers from randomization of the feeder studies. Any patients not entering the BE VITAL OLE were imputed as non-responders, as per EULAR guidance for reporting clinical trial extension data [30]. For each time point up to 2 years, available data are also reported as observed case (OC).

### Ethics approval

Studies were conducted in accordance with the Declaration of Helsinki and the International Conference on Harmonization guidance for Good Clinical Practice. Ethical approval was obtained from the relevant institutional review boards at participating sites and all patients provided written informed consent in accordance with local requirements. Local institutional review board information can be found in Supplementary Appendix S1.

## Results

### Patient disposition and baseline characteristics

Of 852 randomized bDMARD-naïve patients in BE OPTIMAL, 254/281 (90.4%) placebo-randomized and 379/431 (87.9%) bimekizumab-randomized patients completed week 52 and entered BE VITAL; 239/281 (85.1%) placebo-randomized and 359/431 (83.3%) bimekizumab-randomized patients completed to week 104. Of the 400 TNFi-IR patients randomized in BE COMPLETE, 121/133 (91.0%) placebo-randomized patients and 256/267 (95.9%) bimekizumab-randomized patients completed week 16 and entered BE VITAL; 109/133 (82.0%) placebo-randomized patients and 221/267 (82.8%) bimekizumab-randomized patients completed to week 88.

Baseline patient demographics and clinical disease characteristics, reported previously, reflect a patient population with high disease burden (Supplementary Table S2) [23, 31–34]. On average, the time between first PsA diagnosis and baseline was longer in TNFi-IR patients compared with bDMARD-naïve patients. Disease activity was also higher in TNFi-IR patients compared with bDMARD-naïve patients and fewer TNFi-IR patients were employed at baseline.

### Health status

**Figure 1:**
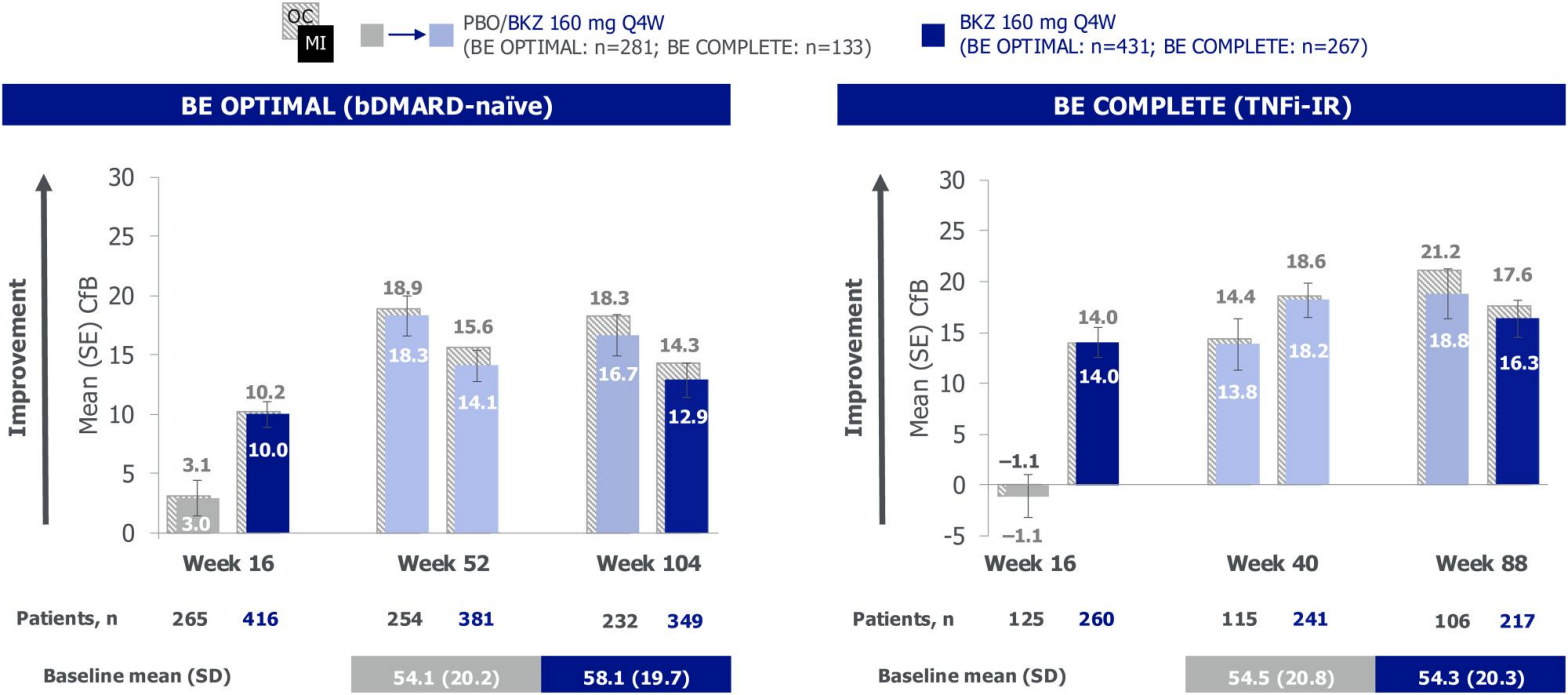
EQ-5D-3L VAS at weeks 16, 52/40 and 104/88 (MI, OC). Randomized set. MI (colored bars) and OC (light grey crosshatched bars) data reported; s.e. reported for MI data only. bDMARD: biologic DMARD; BKZ: bimekizumab; CfB: change from baseline; MI: multiple imputation; OC: observed case; PBO: placebo; Q4W: every 4 weeks; TNFi-IR: tumor necrosis factor inhibitor inadequate response or intolerance; VAS: visual analog scale

At baseline, mean EQ-5D-3L VAS scores were comparable across patient populations and treatment arms [bDMARD-naïve bimekizumab-randomized: 58.1 (s.d. 19.7); placebo-randomized: 54.1 (s.d. 20.2); TNFi-IR bimekizumab-randomized: 54.3 (s.d. 20.3); placebo-randomized: 54.5 (s.d. 20.8); Fig. 1]. Improvements from baseline in EQ-5D-3L VAS scores at week 52/40 [21] were sustained to week 104/88 in bimekizumab-randomized patients, regardless of prior TNFi experience [mean CfB at week 104/88: bDMARD-naïve 12.9 (s.e. 1.4); TNFi-IR 16.3 (s.e. 1.8)]. These translate to percentage improvements from baseline of 24.6% for bDMARD-naïve and 32.4% for TNFi-IR bimekizumab-randomized patients to week 104/88. Similar sustained improvements to week 104/88 were observed in placebo/bimekizumab-randomized patients (Fig. 1).

Improvements from baseline in the EQ-5D-3L utility score achieved by week 52/40 were generally sustained to week 104/88 across treatment arms in both studies (Supplementary Table S3).

### Work productivity

**Figure 2:**
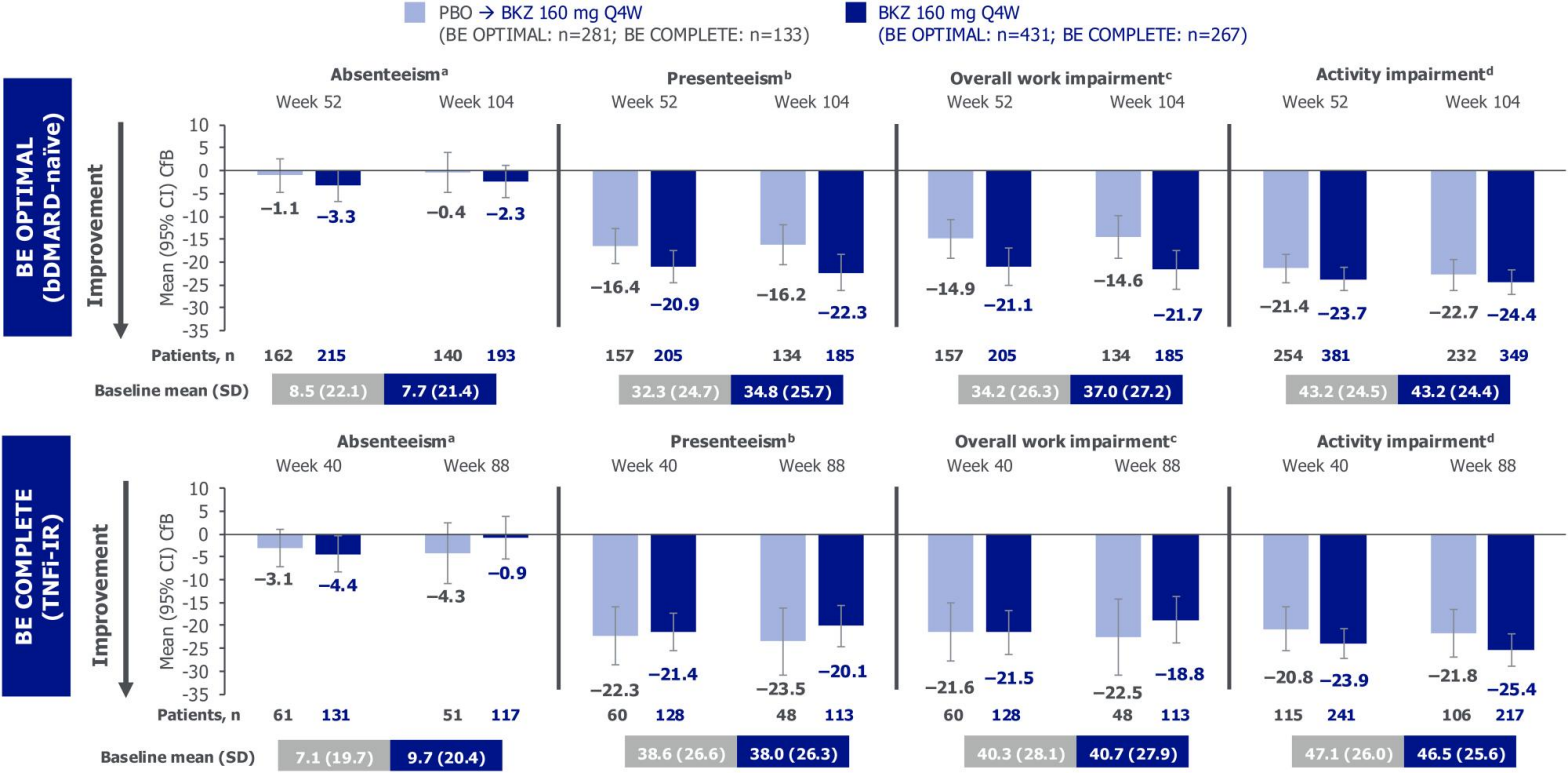
Work productivity CfB at weeks 52/40 and 104/88 (OC). Randomized set. Measured using WPAI-SHP, adapted for PsA. **(a)** Work time missed due to PsA; BE OPTIMAL (PBO-randomized *n* = 189; BKZ-randomized n = 270), BE COMPLETE (PBO-randomized *n* = 75; BKZ-randomized *n* = 162). **(b)** Impairment while working due to PsA; BE OPTIMAL (PBO-randomized *n* = 181; BKZ-randomized *n* = 262), BE COMPLETE (PBO-randomized *n* = 73; BKZ-randomized *n* = 158). **(c)** BE OPTIMAL (PBO-randomized *n* = 181; BKZ-randomized *n* = 262), BE COMPLETE (PBO-randomized *n* = 73; BKZ-randomized *n* = 158). **(d)** Ability to undertake regular, non-work-related activities (e.g. childcare) [26]; BE OPTIMAL (PBO-randomized *n* = 281; BKZ-randomized *n* = 430), BE COMPLETE (PBO-randomized *n* = 133; BKZ-randomized *n* = 267). bDMARD: biologic DMARD; BKZ: bimekizumab; CfB: change from baseline; OC: observed case; PBO: placebo; Q4W: every 4 weeks; TNFi-IR: tumor necrosis factor inhibitor inadequate response or intolerance; WPAI-SHP: Work Productivity and Activity Impairment Questionnaire: Specific Health Problem v2.0

The number of patients with available WPAI-SHP data at baseline and subsequent time points are reported in Supplementary Table S2 and Fig. 2, respectively. At baseline, work impairment was generally numerically higher in TNFi-IR patients compared with bDMARD-naïve patients, as demonstrated by mean scores for absenteeism [bDMARD-naïve bimekizumab-randomized: 7.7 (s.d. 21.4), placebo-randomized: 8.5 (s.d. 22.1); TNFi-IR bimekizumab-randomized: 9.7 (s.d. 20.4), placebo-randomized: 7.1 (s.d. 19.7)], presenteeism [bDMARD-naïve bimekizumab-randomized: 34.8 (s.d. 25.7), placebo-randomized: 32.3 (s.d. 24.7); TNFi-IR bimekizumab-randomized: 38.0 (s.d. 26.3), placebo-randomized: 38.6 (s.d. 26.6)], overall work impairment [bDMARD-naïve bimekizumab-randomized: 37.0 (s.d. 27.2), placebo-randomized: 34.2 (s.d. 26.3); TNFi-IR bimekizumab-randomized: 40.7 (s.d. 27.9), placebo-randomized: 40.3 (s.d. 28.1)] and activity impairment [bDMARD-naïve bimekizumab-randomized: 43.2 (s.d. 24.4), placebo-randomized: 43.2 (s.d. 24.5); TNFi-IR bimekizumab-randomized: 46.5 (s.d. 25.6), placebo-randomized: 47.1 (s.d. 26.0)] (Supplementary Table S2).

In bimekizumab-randomized patients, improvements from baseline in overall work impairment observed at week 52/40 [21] were sustained to week 104/88, for both bDMARD-naïve [mean CfB −21.7 (95% CI −26.0, −17.4)] and TNFi-IR [mean CfB −18.8 (95% CI −23.9, −13.7)] patients, with similar improvements reported in placebo/bimekizumab patients (Fig. 2). Improvements from baseline in presenteeism were also sustained to week 104/88 for both bDMARD-naïve [mean CfB −22.3 (95% CI −26.3, −18.4)] and TNFi-IR [mean CfB −20.1 (95% CI −24.6, −15.6)] bimekizumab-randomized patients. Similar improvements were observed for activity impairment (Fig. 2). Improvements from baseline in absenteeism were generally sustained to week 104/88 in bimekizumab-randomized bDMARD-naïve [mean CfB −2.3 (95% CI −5.8, 1.3)] and TNFi-IR patients [mean CfB −0.9 (95% CI −5.5, 3.7)].

**Figure 3:**
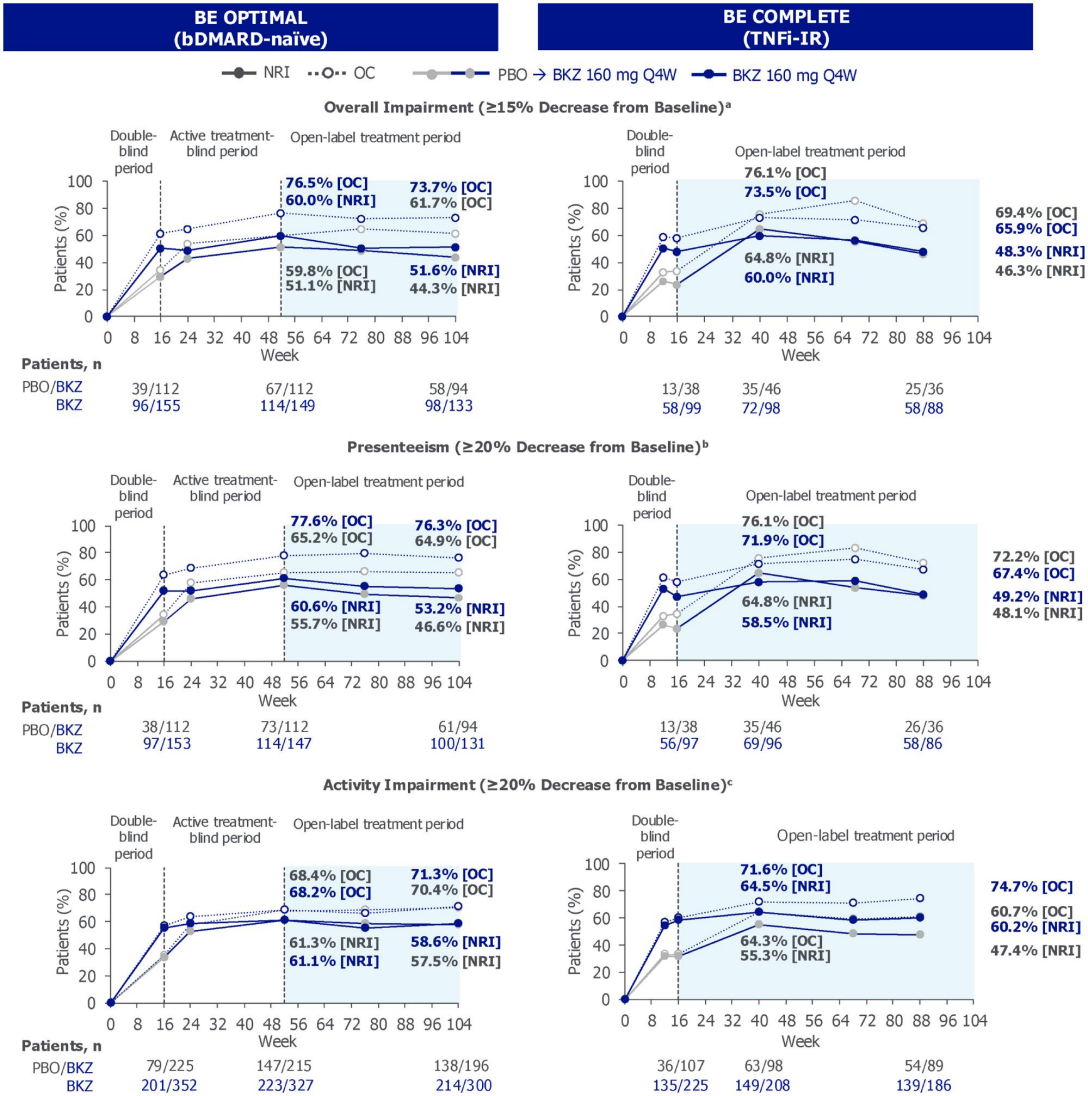
Work productivity MCID responders to week 104/88 (NRI, OC). Randomized set. **(a)** In patients with ≥15% overall work productivity impairment at baseline (BE OPTIMAL PBO/BKZ *n* = 131, bimekizumab *n* = 190; BE COMPLETE PBO/BKZ *n* = 54, BKZ *n* = 120). **(b)** In patients with ≥20% work impairment at baseline (BE OPTIMAL PBO/BKZ *n* = 131, BKZ *n* = 188; BE COMPLETE PBO/BKZ *n* = 54, BKZ *n* = 118). **(c)** In patients with ≥20% activity impairment at baseline (BE OPTIMAL PBO/BKZ *n* = 240, BKZ *n* = 365; BE COMPLETE PBO/BKZ *n* = 114, BKZ *n* = 231). bDMARD: biologic DMARD; BKZ: bimekizumab; MCID: minimal clinically important difference; NRI: non-responder imputation; OC: observed case; PBO: placebo; Q4W: every 4 weeks; TNFi-IR: tumor necrosis factor inhibitor inadequate response or intolerance

Among bimekizumab-randomized patients with ≥15% overall work impairment at baseline, similar proportions of bDMARD-naïve [98/190 (51.6%)] and TNFi-IR [58/120 (48.3%)] patients achieved an MCID in overall work impairment at week 104/88; similar trends were seen in placebo/bimekizumab patients (Fig. 3). Similar trends were observed across the other WPAI-SHP domains to week 104/88, regardless of prior TNFi experience.

**Figure 4:**
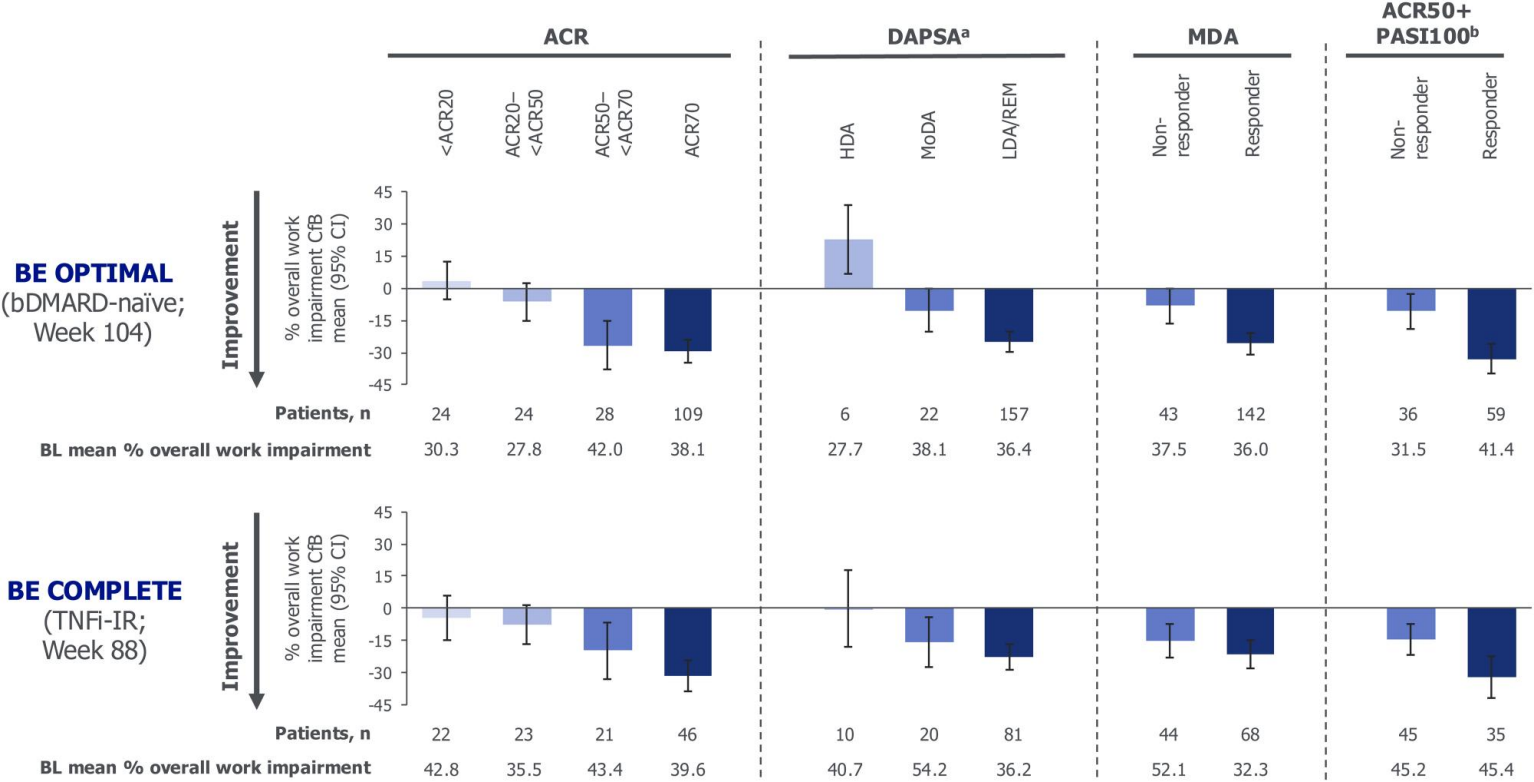
Association of disease control criteria with percentage overall work impairment at week 104/88 for bimekizumab-randomized patients (OC). Randomized set. Assessed in employed patients. Measured using WPAI-SHP adapted for PsA. Categories are mutually exclusive. **(a)** DAPSA HDA: >28; MoDA: >14–≤28; LDA/REM: ≤14; **(b)** in patients with psoriasis affecting ≥3% BSA at baseline. bDMARD: biologic DMARD; BL: baseline; BSA: body surface area; CfB: change from baseline; DAPSA: Disease Activity Index for Psoriatic Arthritis; HDA: high disease activity; LDA: low disease activity; MDA: minimal disease activity; MoDA: moderate disease activity; OC: observed case; PASI: Psoriasis Area and Severity Index; REM: remission; TNFi-IR: inadequate response or intolerance to tumor necrosis factor inhibitor; WPAI-SHP: Work Productivity and Activity Impairment Questionnaire: Specific Health Problem v2.0

At week 104/88, both bDMARD-naïve and TNFi-IR patients achieving ACR70 demonstrated greater improvements in overall work impairment *vs* those with non-response (<ACR20) in both bDMARD-naïve [mean CfB −29.4 (95% CI −34.9, −23.9) *vs* 3.6 (95% CI −5.4, 12.6), respectively] and TNFi-IR patients [mean CfB −31.5 (95% CI −39.0, −24.1) *vs* −4.4 (95% CI −14.8, 6.0), respectively] (Fig. 4). Similar trends were observed for patients who achieved ACR50 but not ACR70, such that these patients demonstrated greater improvements in overall work impairment *vs* non-responders.

Similarly, greater improvements in overall work impairment at week 104/88 were observed in patients achieving DAPSA LDA/REM *vs* those with HDA [mean CfB: bDMARD-naïve −25.0 (95% CI −29.6, −20.4) *vs* 23.1 (95% CI 7.1, 39.1); TNFi-IR −22.6 (95% CI −28.6, −16.6) *vs* −0.1 (95% CI −18.1, 17.9), respectively] (Fig. 4).

At week 104/88, patients achieving MDA demonstrated greater improvements in overall work impairment *vs* non-responders in both bDMARD-naïve [mean CfB: −25.8 (95% CI −30.7, −20.9) *vs* −8.2 (95% CI −16.5, 0.1), respectively] and TNFi-IR patients [mean CfB: −21.5 (95% CI −28.1, −14.8) *vs* −15.3 (95% CI −23.3, −7.3), respectively] (Fig. 4).

Similarly, greater improvements from baseline in overall work impairment to week 104/88 were observed in ACR50 + PASI100 responders *vs* non-responders in both bDMARD-naïve [mean CfB −32.9 (95% CI −39.8, −26.0) *vs* −10.7 (95% CI −18.9, −2.4)] and TNFi-IR [mean CfB −32.0 (95% CI −41.7, −22.4) *vs* −14.6 (95% CI −21.6, −7.6), respectively] patients with psoriasis affecting ≥3% BSA at baseline (Fig. 4).

Similar trends were observed across the other WPAI-SHP domains, regardless of prior TNFi experience (Supplementary Fig. S2).

### Cost analysis

**Figure 5:**
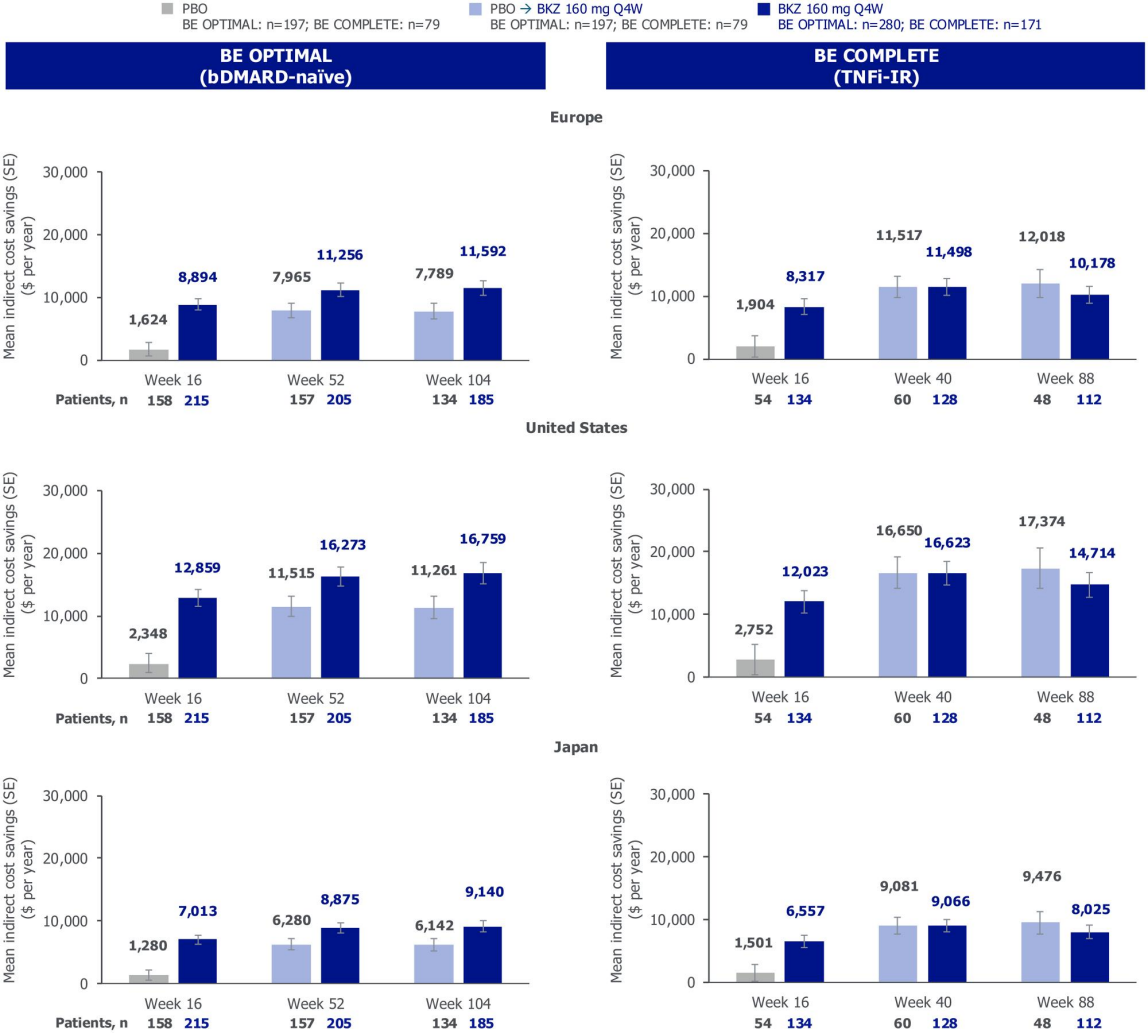
Annualized estimated indirect cost savings from overall work impairment at weeks 16, 52/40 and 104/88 (OC). Randomized set. Assessed in employed patients. bDMARD: biologic DMARD; BKZ: bimekizumab; OC: observed case; PBO: placebo; Q4W: every 4 weeks; TNFI-IR: tumor necrosis factor inhibitor inadequate response or intolerance

Annualized indirect cost savings in US dollars from improved overall work impairment across both studies and treatment arms in Europe, the USA and Japan are reported in Fig. 5. In both bDMARD-naïve and TNFi-IR patients, indirect cost savings at week 16 were numerically greater in bimekizumab-randomized patients *vs* placebo-randomized patients across all three regions [bDMARD-naïve: Europe (US$8894 *vs* US$1624), USA (US$12 859 *vs* US$2348), Japan (US$7013 *vs* US$1280); TNFi-IR: Europe (US$8317 *vs* US$1904), USA (US$12 023 *vs* US$2752), Japan (US$6557 *vs* US$1501)].

Regardless of prior TNFi experience, there was an increase in indirect cost savings between week 16 and week 52/40 in Europe (bDMARD-naïve: US$8894 *vs* US$11 256; TNFi-IR: US$8317 *vs* US$11 498), the USA (bDMARD-naïve: US$12 859 *vs* US$16 273; TNFi-IR: US$12 023 *vs* US$16 623) and Japan (bDMARD-naïve: US$7013 *vs* US$8875; TNFi-IR: US$6557 *vs* US$9066). These indirect cost savings were sustained to week 104/88 among bDMARD-naïve and TNFi-IR patients.

Additionally, in bDMARD-naïve patients, indirect cost savings from improved overall work impairment continued to be numerically greater in bimekizumab-randomized patients *vs* placebo-randomized patients to week 104 in Europe (US$11 592 *vs* US$7789), the USA (US$16 759 *vs* $11 261) and Japan (US$9140 *vs* US$6142).

Indirect cost savings at week 88 for TNFi-IR bimekizumab-randomized patients and bimekizumab/placebo were generally similar (Europe, US$10 178 *vs* US$12 018; the USA, US$14 714 *vs* US$17 374; Japan, US$8025 *vs* US$9476).

## Discussion

Treatment with bimekizumab resulted in sustained improvements in health status and work productivity up to 2 years in patients with PsA. Notably, achieving stringent disease control criteria was associated with the greatest improvements in work productivity, particularly in overall work impairment, presenteeism and activity impairment. These improvements were also associated with high estimated annualized indirect cost savings from the reduction in overall work impairment.

Improvements in health status, assessed using the EQ-5D-3L, were sustained to week 104/88 in bDMARD-naïve and TNFi-IR patients. The EQ-5D-3L provides a holistic overview of health, relevant in measuring PsA’s impact on physical, emotional and social functioning symptoms [35]. These long-term improvements complement findings from previous publications that report substantial improvements in other patient-reported outcomes, sustained up to 2 years [20].

Bimekizumab treatment in bDMARD-naïve and TNFi-IR patients with PsA demonstrated improvements in overall work impairment, presenteeism and activity impairment that were sustained to week 104/88; these findings extend previously published improvements in work productivity up to 1 year [21].

Furthermore, high proportions of patients with PsA achieved an MCID in overall work impairment, presenteeism and activity impairment to week 104/88, highlighting the potential for bimekizumab treatment to improve long-term employment outcomes and reduce work disability among patients with PsA.

Achieving stringent thresholds of disease control reduces the burden on patients’ productivity in the workplace and household [36, 37]. Here it was demonstrated that achieving more stringent disease control criteria (MDA, ACR50 + PASI100, ACR50, DAPSA LDA/REM) was associated with greater improvements in WPAI-SHP domains.

Mean values for absenteeism scores at baseline were low across both studies (bDMARD-naïve bimekizumab-randomized: 7.7, placebo-randomized: 8.5; TNFi-IR bimekizumab-randomized: 9.7, placebo-randomized: 7.1) and, of note, smaller CfBs were observed for this domain to week 104/88 compared with the others. This trend, in which improvements in absenteeism are generally smaller than those in other WPAI-SHP domains, has been commonly reported in other randomized controlled trails in patients with PsA [26, 28, 38]. Further investigation into the contributing factors may be warranted. The smaller CfBs may have also contributed to the less pronounced associations seen between achievement of stringent disease control criteria and reductions in absenteeism. Nonetheless, the overall trend remained consistent with that observed across the other WPAI-SHP domains.

Notably, patients with psoriasis affecting ≥3% BSA at baseline achieving stringent disease control in musculoskeletal and skin domains (ACR50 + PASI100) generally experienced the greatest improvements in overall work impairment at week 104/88 across both studies.

Overall, these results suggest that high levels of disease control associated with bimekizumab treatment contribute to greater improvements in general health and work productivity. These findings align with key recommendations from the EULAR and the Group for Research and Assessment of Psoriasis and Psoriatic Arthritis guidelines to prioritize improvement in HRQoL in patients with PsA [33, 39]. Additionally, the ability to work and the ability to engage in social participation are frequently reported as a priority by patients with PsA [40].

PsA has a high cost for society, with annual direct healthcare-related costs reported to be as high as US$1.9 billion, with indirect costs estimated to be even greater, accounting for 52–72% of total disease costs [11]. There were high estimated yearly indirect cost savings from improved overall work impairment with bimekizumab treatment as early as week 16, sustained up to week 104/88 in Europe, the USA and Japan. Indirect cost savings associated with other treatments for PsA have been reported in a meta-analysis and in individual studies examining longer-term outcomes [7, 28]. While these reports indicate positive cost savings, direct comparisons cannot be made due to differences in study design, patient populations, methods and calculation years [7, 28].

### Strengths

The current analysis uses health status and work productivity data collected over a longer-term period in BE OPTIMAL and BE COMPLETE, which allows the findings to complement efficacy, safety and other patient-reported outcome evidence reported in previous publications [20, 21]. These findings also demonstrated the association between achieving stringent disease control criteria, including the composite endpoints MDA, DAPSA and ACR50 + PASI100, and improvement in overall work productivity, thereby providing further support for using treatments with demonstrated long-term efficacy that address multiple PsA disease domains.

Notably, sustained improvements in health status and work productivity and yearly indirect work impairment–related cost savings were consistent between bDMARD-naïve and TNFi-IR patients up to 2 years, the latter representing a difficult-to-treat population in whom achieving efficacy outcomes is more difficult [34].

The EQ-5D-3L utility score was assessed using a UK tariff, which is commonly used in other, similar studies, allowing for comparability across findings [7].

### Limitations

The OLE study BE VITAL enrolled patients from both the BE OPTIMAL and BE COMPLETE studies. In BE OPTIMAL, patients received open-label treatment after week 52; in BE COMPLETE, patients received open-label treatment after week 16. However, studies were conducted over the same time period at overlapping study sites and countries, helping to ensure consistency in study conditions.

Generalizability of these data are limited when considering patients in the clinical setting; patients in real-world settings may experience greater disease heterogeneity, comorbidities and variations in treatment adherence, which can affect clinical outcomes.

Assessment of the EQ-5D-3L utility score using a UK tariff may be considered a limitation, as it is country specific.

The current analysis does not consider the nature of employment. For example, higher work disability is associated with manual work in patients with PsA [41]. Self-selection towards non-manual labour may have occurred, leading to a potential underestimation of work impairment; patients with PsA may have been unable to perform their preferred manual labour work and therefore changed to a non-preferred, non-manual role in the workplace. The current analysis does not consider the underlying reasons for the gain or loss of employment, including whether it is attributable to PsA.

The economic data analysis involves numerous underlying assumptions. OECD national accounts wages may differ from wages from alternative sources as a result of their broader worker coverage, given that they include both full-time and part-time workers at a full-time equivalent rate [29]. This methodology is likely to lower average wages as compared with wage estimates that consider full-time workers only [29]. Additionally, national accounts wages include low-paid apprentices and employees in part-time jobs who may be excluded from other surveys, further reducing average wage levels in some countries [29]. Finally, national accounts wages include average earnings from secondary jobs of workers with two or more jobs, which can increase wage estimates; however, this effect is usually small and varies by country [29].

### Future research

Future publications will report longer-term data to show how these results are sustained in the context of a chronic disease requiring lifelong treatment.

## Conclusion

Treatment with bimekizumab was associated with sustained improvements in standard measures of health status and sustained clinically meaningful improvements in work productivity, including presenteeism, overall work impairment and activity impairment, up to 2 years, which could contribute to indirect cost savings for patients and society overall. Improvements were consistent between bDMARD-naïve and TNFi-IR patients.

## Supplementary Material

rkag009_Supplementary_Data

## Data Availability

Data from this article may be requested by qualified researchers 6 months after product approval in the USA and/or Europe, or global development is discontinued, and 18 months after trial completion. Investigators may request access to anonymized individual patient data and redacted study documents that may include raw datasets, analysis-ready datasets, study protocol, blank case report form, annotated case report form, statistical analysis plan, dataset specifications and clinical study report. Prior to use of the data, proposals need to be approved by an independent review panel at https://vivli.org/ and a signed data sharing agreement will need to be executed. All documents are available in English only, for a prespecified time, typically 12 months, on a password-protected portal.
